# Immunometabolism in *Caenorhabditis elegans*

**DOI:** 10.1371/journal.ppat.1008897

**Published:** 2020-10-08

**Authors:** Sarah M. Anderson, Read Pukkila-Worley

**Affiliations:** Program in Innate Immunity, Division of Infectious Diseases and Immunology, University of Massachusetts Medical School, Worcester, Massachusetts, United States of America; University of Massachusetts, Worcester, UNITED STATES

Host metabolism is profoundly altered during bacterial infection, both as a consequence of immune activation and secondary to virulence strategies of invading pathogens. As a result, the metabolic pathways that regulate nutrient acquisition, energy storage, and resource allocation in host cells must adapt to pathogen stress in order to meet the physiological demands of the host during infection. However, the specific alterations in host metabolism that occur during bacterial infection are challenging to decipher, owing to physiological disruption in multiple organ systems that occur during infection and complex metabolic interactions between the host and the pathogen. In this regard, the nematode *Caenorhabditis elegans* has emerged as a useful starting point to characterize fundamental principles of immunometabolism. For nematodes, bacteria are both a source of food and agents of disease. As such, *C*. *elegans* has evolved innate immune defenses coordinated by intestinal epithelial cells, which promote survival during infection by ingested pathogens. Studies of pathogen infection in *C*. *elegans* can therefore be used to define changes in host metabolism specifically associated with infection by pathogenic bacteria in the intestine. Here, we discuss 5 concepts that have emerged in studies of metabolic and immune interactions in *C*. *elegans* (Fig 1). The major emerging theme is that the immune response and the ability to survive pathogen infection is heavily influenced by pathogen-induced changes in host metabolism.

**Fig 1:**
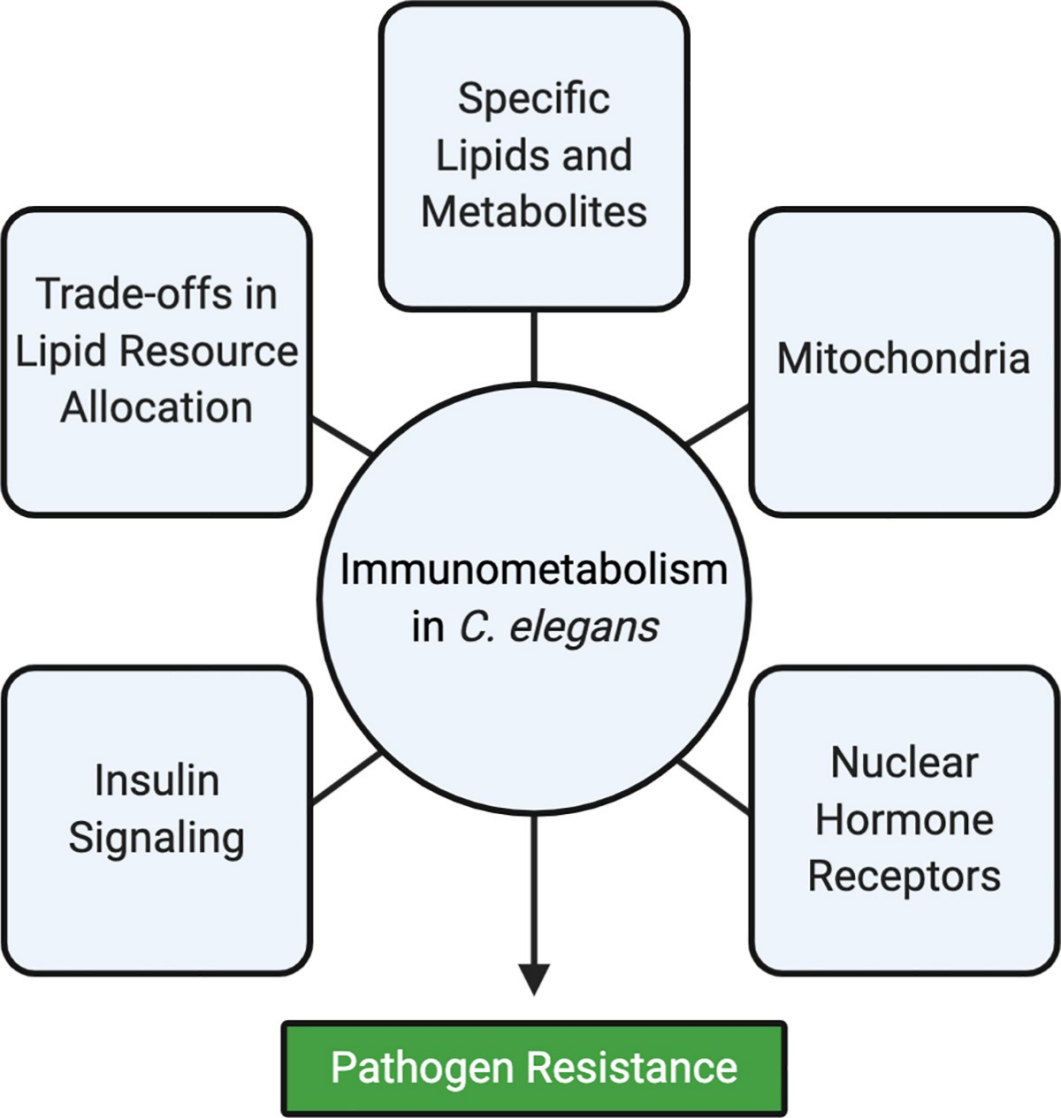
Immunometabolism in *C*. *elegans*. A schematic diagram presents 5 concepts that have emerged in studies of metabolic and immune interactions in *C*. *elegans*.

## Insulin signaling integrates host metabolism, pathogen resistance, and longevity

The insulin/insulin-like growth factor signaling pathway integrates host nutritional status and environmental cues to control core physiological processes in *C*. *elegans*, including metabolism, growth rate, behavior, and stress resistance [1]. Activation of the *C*. *elegans* insulin/IGF-1 transmembrane receptor (IGFR) ortholog DAF-2 by insulin-like peptides results in the phosphorylation of the Foxo transcription factor DAF-16, causing it to be sequestered in the cytoplasm [2]. Low activity of DAF-2 allows DAF-16 to translocate to the nucleus where it controls the transcriptional output of this pathway [3]. Constitutive activation of DAF-16 in *daf-2* loss-of-function mutants extends nematode life span up to 3 times than that of wild-type animals and drives resistance to both abiotic stresses and pathogen infection [4,5]. In addition, de novo lipogenesis is increased in *daf-2* mutants, which leads to accumulation of somatic fat. The pathogen resistance and life span extension phenotype of *daf-2* mutants require the p38 mitogen-activated protein kinase (MAPK) PMK-1 pathway, a critical innate immune pathway in *C*. *elegans* [6]. However, the transcriptional targets of the DAF-2/DAF-16 and the p38 MAPK PMK-1 pathways during pathogen infection have essentially no overlap, suggesting that these pathways operate in parallel to promote resistance to pathogen infection [6]. Interestingly, infection by the bacterial pathogen *Pseudomonas aeruginosa* activates DAF-2 signaling as an offensive mechanism to suppress host immune defenses by causing DAF-16 to be sequestered in the cytoplasm [7]. Insulin/insulin-like growth factor signaling is strongly conserved across metazoan evolution. Thus, examination of the mechanisms by which the DAF-2/DAF-16 pathway integrates information about host nutrition to control metabolism, pathogen resistance, and life span may yield fundamental insights about immunometabolism.

## Allocation of lipid resources affects physiological trade-offs between pathogen resistance, life span, and reproduction

Studies of immunometabolism in *C*. *elegans* have uncovered trade-offs between immune activation and lipid homeostasis that affect pathogen resistance, reproduction, and life span. The cytoprotective transcription factor SKN-1, the *C*. *elegans* ortholog of mammalian Nrf2, coordinates transcriptional responses that restore cellular homeostasis during oxidative, proteotoxic, and metabolic stresses and also provides protection during pathogen infection [8,9]. During bacterial infection, activation of SKN-1 promotes resistance to pathogen-derived toxins and drives redistribution of fat from the soma to the germline [10]. However, altered lipid homeostasis in *C*. *elegans* with unchecked SKN-1 activation has lasting deleterious effects, which impair organismal health later in life [10]. Thus, the activity of SKN-1 is closely regulated, in part through epigenetic modifications, which redirect its transcriptional output to meet the physiological need [10].

In addition, pathogen and stress-resistance programs are suppressed as animals increase resource investment to promote reproductive success. Two conserved homeodomain transcription factors CEH-60/PBX and UNC-62/MEIS function as a heterodimer to promote the synthesis of lipoproteins, which shuttle lipids to the germline to support embryogenesis. In addition, the CEH-60:UNC-62 complex also suppresses genes, which are important for pathogen defense and longevity [11]. Similarly, TCER-1, a transcription elongation and splicing factor, promotes reproductive fitness and lipid synthesis at the expense of pathogen and abiotic stress defenses [12]. Finally, a core host defense pathway in *C*. *elegans*, the p38 MAP PMK-1 pathway, is activated by nutrient signals independently of canonical mechanisms that sense food availability and accelerates aging when it is aberrantly induced, providing an example of the deleterious effects of immune activation on longevity [13].

## Lipid metabolism is required for immune activation and pathogen defense

*C*. *elegans* pathogenesis assays have defined requirements for specific lipids and lipogenesis enzymes in innate immune regulation and pathogen defense. *C*. *elegans* can synthesize the full range of fatty acid molecules de novo and thus does not have a dietary requirement for specific fatty acids. Monounsaturated and polyunsaturated fatty acids are synthesized through sequential action of conserved elongase (*elo*) and desaturase (*fat*) genes. The Δ6-desaturase *fat-3*, which produces the polyunsaturated fatty acids gamma-linoleic acid and stearidonic acid, is required for the basal expression of innate immune genes and resistance to infection by the bacterial pathogen *P*. *aeruginosa* [14]. In addition, the 2 stearoyl-coenzyme A desaturases that synthesize the monounsaturated fatty acid oleate in *C*. *elegans*, *fat-6* and *fat-7*, are required for the induction of innate immune genes [15]. Accordingly, nematodes with loss-of-function mutations in *fat-6* and *fat-7* are hypersusceptible to infection by diverse pathogens, which can be rescued by the addition of exogenous oleate [15].

Additionally, low levels of s-Adenosylmethionie (SAM), the methyl donor that modifies nucleic acids and histones and is involved in producing phospholipids, result in a decrease in phosphatidylcholine (PC). Low levels of PC in animals that lack *sams-1*, an enzyme that produces SAM, induce expression of lipogenesis genes resulting in lipid droplet accumulation [16,17]. Interestingly, low PC increases the basal expression of immune genes in *C*. *elegans* feeding on nonpathogenic food. However, low levels of activating histone methylation in these animals also limit pathogen-responsive transcription and renders animals more susceptible to infection [16].

Together, these studies in *C*. *elegans* reveal novel connections between nutrient stores, metabolism, and host susceptibility to bacterial infection.

## Mitochondria link energy metabolism and immune activation

Mitochondria are required for multiple aspects of cellular metabolism. Bacteria often target mitochondria during infection as an offensive strategy to promote tissue damage. For example, *P*. *aeruginosa* secretes phenazine toxins, electron shuttles that disrupt mitochondrial function, [18] and *Streptomyces* sp. elaborate antimycin A and oligomycin, inhibitors of mitochondrial respiration that are widely used in the laboratory. Studies in *C*. *elegans* have characterized several host countermeasures that have evolved to detect mitochondrial dysfunction as a sign of pathogen infection.

The unfolded protein response in mitochondria (UPR^mt^) is regulated by the transcription factor ATFS-1, a unique protein that contains both a nuclear localization (NLS) and a mitochondrial targeting sequence (MTS) [19,20]. Healthy mitochondria import ATFS-1 efficiently, but during mitochondrial dysfunction, protein import is impaired, and ATFS-1 accumulates in the cytoplasm, where it can traffic to the nucleus via its NLS. ATFS-1 activates a transcriptional program in the nucleus that promotes both recovery of mitochondrial function and defense against pathogen infection through the induction of secreted innate immune effectors [19,20]. Interestingly, the pathogen *P*. *aeruginosa* evolved mechanisms to suppress the UPR^mt^ by exploiting a host pathway that negatively regulates ATFS-1 [18].

In addition, ceramides, a class of host lipids, protect *C*. *elegans* from mitochondrial dysfunction induced by toxins or pathogen exposure [21]. Likewise, disruption of mitochondrial function activates the nuclear hormone receptor *nhr-45*, which induces detoxification programs that provide protection during pathogen infection [22]. Finally, the iron-binding siderophore pyoverdine, which is produced by *P*. *aeruginosa*, causes mitochondrial dysfunction during infection, which engages protective destruction of damaged mitochondria (mitophagy) [23].

Together, these studies demonstrate that mitochondrial function is closely guarded by host surveillance pathways that function to restore homeostasis and activate protective innate immune defenses.

## Transcriptional control of innate immunity and metabolism by conserved nuclear hormone receptors

The *C*. *elegans* genome encodes a large number of nuclear hormone receptors (NHRs), unique transcription factors that program adaptive transcriptional responses following recognition of specific ligands, such as fatty acids, metabolites, hormones, and xenobiotics. NHRs regulate a number of basic biological processes in *C*. *elegans*, including lipid and cholesterol metabolism, life span, development, and anti-pathogen defenses. The marked expansion of NHRs in nematodes—284 NHRs are present in *C*. *elegans*, whereas Drosophila and humans have only 21 and 48, respectively—suggests that these proteins may play particularly important roles in nematode physiology, such as the integration of host metabolism with innate immunity to promote resistance to pathogen infection.

Interestingly, 264 of the 284 NHRs in the *C*. *elegans* genome are orthologous to the alpha isoform of the mammalian nuclear receptor hepatocyte nuclear factor 4 (HNF4⍺). HNF4⍺ is expressed in the intestinal epithelium and in hepatocytes and has been implicated in the control of intestinal inflammation and the pathogenesis of inflammatory bowel disease and cancer. In *C*. *elegans*, the HNF4⍺ homolog NHR-86 surveys the chemical environment to activate protective anti-pathogen defenses by binding to the promoters of immune effector genes [24]. These data suggest that the expansion of the HNF4⍺ family in *C*. *elegans* may have been fueled, at least in part, by the roles of these proteins in the activation of host defense responses. In addition, the *C*. *elegans* homolog of peroxisome proliferator-activated receptor (PPAR), NHR-49, a central regulator of fat metabolism, is required for resistance to multiple gram-positive bacteria, including *Enterococcus faecalis* [25]. Of note, NHR-49 interacts with a conserved subunit of the Mediator complex MDT-15/MED15 to control the production of fatty acids, and a separate study found that MDT-15 also coordinates immune defenses during pathogen infection [26]. Thus, NHR-49 and MDT-15 regulation of fatty acid metabolism may support immune function in nematodes. In addition, the *C*. *elegans* homolog of the liver X receptor (LXR), NHR-8, which controls cholesterol and bile acid homeostasis, is required for defense against infection with *P*. *aeruginosa* [27,28]. Finally, the nuclear hormone receptor NHR-14 links iron availability with the induction of innate immune defenses that provide protection from pathogen infection [29].

In summary, NHRs are able to mount rapid transcriptional responses to specific intracellular and extracellular cues and are thus poised to integrate host physiology and metabolism to provide protection from pathogens during infection. Future studies of the mechanisms by which NHRs control immunometabolism in *C*. *elegans* are of particular interest.
